# Feeding Problems Are Persistent in Children with Severe Congenital Hyperinsulinism

**DOI:** 10.3389/fendo.2016.00008

**Published:** 2016-02-09

**Authors:** Indraneel Banerjee, Lynette Forsythe, Mars Skae, Hima Bindu Avatapalle, Lindsey Rigby, Louise E. Bowden, Ross Craigie, Raja Padidela, Sarah Ehtisham, Leena Patel, Karen E. Cosgrove, Mark J. Dunne, Peter E. Clayton

**Affiliations:** ^1^Department of Paediatric Endocrinology, Royal Manchester Children’s Hospital, Manchester, UK; ^2^Faculty of Medical and Human Sciences, University of Manchester, Manchester, UK; ^3^Department of Dietetics and Nutrition, Royal Manchester Children’s Hospital, Manchester, UK; ^4^Department of Paediatric Surgery, Royal Manchester Children’s Hospital, Manchester, UK; ^5^Faculty of Life Sciences, University of Manchester, Manchester, UK

**Keywords:** glucose, insulin, hypoglycemia, congenital hyperinsulinism, feeding, feeding problems

## Abstract

**Background:**

Congenital hyperinsulinism (CHI) is a rare but severe disorder of hypoglycemia in children, often complicated by brain injury. In CHI, the long-term prevention of hypoglycemia is dependent on reliable enteral intake of glucose. However, feeding problems (FPs) often impede oral glucose delivery, thereby complicating the management of hypoglycemia. FPs have not been systematically characterized in follow-up in a cohort with CHI.

**Aims:**

We aimed to determine the prevalence, types, and persistence of FPs in a cohort of children with CHI and investigate potential causal factors.

**Methods:**

FPs were defined as difficulty with sucking, swallowing, vomiting, and food refusal (or a combination) in an observational study in 83 children in a specialized CHI treatment center. The prevalence of FPs at diagnosis, 6, and 12 months after diagnosis were noted. Genetic mutation status and markers of severity of CHI were tested for association with FPs.

**Results:**

A third of children with CHI had FPs (*n* = 28), of whom 93% required antireflux medication and 75% required nasogastric and gastrostomy tube feeding. Sucking and swallowing problems were present at diagnosis but absent later. Vomiting was present in 54% at 6 months, while food refusal was present in 68% at 6 months and 52% at 12 months. The age at commencing and stopping nasogastric tube feeding did not correlate with FPs frequency at 6 and 12 months. Children with FPs had severe hypoglycemia at diagnosis and required glucagon infusion more often [odds ratio (OR) (95% confidence intervals) (95% CI) 28.13 (2.6–300.1), *p* = 0.006] to normalize glucose levels. FPs were more frequent in those with diffuse CHI undergoing subtotal pancreatectomy [*n* (%) = 10 (35%) vs. 0 (0%), *p* < 0.001], in contrast to those with spontaneous resolution [6 (22%) vs. 32 (58%), *p* = 0.002]. Those undergoing focal lesionectomy also had reduced FPs at 6 months after diagnosis [OR (95% CI) 0.01 (0.0–0.2), *R*^2^ = 0.42, *p* = 0.004]. These observations suggest that persistence of hyperinsulinism was associated with FPs.

**Conclusion:**

FPs occur in a significant proportion of children with CHI. Severe hyperinsulinism, rather than nasogastric tube feeding or medications, is the main factor associated with FPs.

## Introduction

Congenital hyperinsulinism (CHI) is a rare condition of hypoglycemia (reported incidence of severe CHI, 1:30,000–1:50,000) due to unregulated and excessive insulin secretion with the potential to cause hypoglycemia-related brain injury (1, 2). Children diagnosed with CHI can be significantly ill, often requiring prolonged periods of hospitalization and intensive medical treatment to achieve glycemic stability (3). They are often intolerant of oral feeding and may require parenteral nutrition to ensure adequate carbohydrate intake to prevent hypoglycemia. Feeding problems (FPs) are common in many seriously ill children, but are particularly frequent and complex in children with CHI, a feature that has been recognized in case reports (4, 5), and in a recent retrospective observational study (6). However, the prevalence, types, extent, and natural history of FPs in follow-up assessments have not been described in children with CHI.

Children with CHI often require treatment with oral diazoxide, which may be unpleasant to taste. In those who suffer adverse effects, or are unresponsive to diazoxide, subcutaneous octreotide, a somatostatin analog is used as second-line treatment. Unfortunately, octreotide may cause gastrointestinal dysmotility and thereby aggravate FPs. The relative contribution of various factors in CHI, including severity of hyperinsulinism, iatrogenic adverse effects from medication, and gastrostomy feeding, to FPs in CHI are not known. Ensuring adequate feeding tolerance to prevent recurrent hypoglycemia, particularly at discharge from hospital, is an essential part of the long-term clinical management of CHI. We have therefore investigated the nature and duration of FPs and determined possible causal factors in a contemporary cohort of CHI patients with the following objectives:

1. To investigate the prevalence and types of FPs in a cohort of children with CHI at diagnosis and in follow-up assessments at 6 and 12 months.
2. To determine factors that could be associated with persistent FPs in this cohort.

## Materials and Methods

FPs were identified at diagnosis in a cohort of children (*n* = 83) with CHI presenting with severe hypoglycemia, consecutively over a period of 4 years (2008–2011) to a specialized referral treatment center for CHI. The design of the study was observational over a 12-month period. Diagnosis of CHI was confirmed by biochemical investigations as described in a previous study (7). Children presenting with significant perinatal asphyxia (i.e., those showing clinical features suggestive of hypoxic ischemic encephalopathy), surgical bowel anomalies, genetic syndromes, and severe neurological abnormalities precluding feeding were excluded from recruitment. However, assessment for mild neurodevelopmental impairment was not performed. Each child with FPs was reviewed at 6 and 12 months after diagnosis. Height and weight measurements at these intervals were converted to Standard Deviation Scores (SDS) using UK-based normative data (8) and were analyzed by non-parametric tests for related samples, testing for differences between follow-up visits. All children in this cohort had received diazoxide, at least initially, for the treatment of hypoglycemia. The standard feeding practice for children with CHI over the time period of the study comprised of oral milk/food intake, which was increased in 4–6 hourly steps as tolerated.

Feeding problems were diagnosed by a dietitian and a speech and language therapist within 14 days of diagnosis of CHI and at 6 and 12 months at routine follow-up clinic visits with multidisciplinary input. In the neonatal period, the assessment of FPs was made at the time of establishing oral feeding irrespective of the use of intravenous fluids or medications, each feed being at least 15 ml in volume. FPs were categorized into sucking problems, swallowing problems, vomiting, and food refusal. Sucking and swallowing problems were diagnosed objectively, if the child did not demonstrate adequate oral and pharyngeal reflexes when offered a teat, from either a breast or a bottle on consecutive occasions for at least 2 days. If sucking and swallowing problems were recurrent, and enteral feeding was deemed clinically important to maintain normal blood glucose levels, a nasogastric tube was inserted. Children who received this tube were offered oral feeding first with top-up milk feeds if unable to finish the complete prescribed volume of milk. The tube was withdrawn if satisfactory oral feeding was established for more than 24 h. The tube was also removed in those children receiving a percutaneous-feeding gastrostomy.

Vomiting was considered as a FPs by subjective assessment of the dietitian, if more than 50% of food contents were brought up for at least 50% of feeds offered to the child. In the absence of known anatomical causes, such as precipitating and vomiting, gastroesophageal reflux was assumed as the etiology. To treat vomiting, ranitidine, feed thickener, and domperidone were trialed in that order, depending on the response. Food refusal was considered as a FPs if the child did not demonstrate an interest to engage in oral-feeding maneuvers for milk feeds in the neonatal period and liquid and solid foods in later life, in the absence of sucking and swallowing problems. Those who did not have FPs around the time of diagnosis received routine follow-up as clinically indicated. However, as these children did not develop FPs in later follow-up, they were not assessed specifically at 6 and 12 months, unlike the FPs cohort.

Children and families in the FPs cohort required clinical psychology support, based on clinical need. However, formal psychological assessment was not performed.

All children in the cohort were screened for the presence of genetic mutations and received clinical management depending on investigation algorithms described previously (7). Focal CHI was diagnosed after 18 fluoro Dopa PET-CT scanning and confirmed by histology examination after pancreatectomy (3). The maximum dose of diazoxide (milligram per kilogram per day) and the need for a glucagon infusion were considered as surrogate markers for the severity of CHI at diagnosis (9). Carbohydrate requirement was not available in children with late presenting CHI and therefore was not used as a marker of severity. Children were considered to have spontaneous resolution if medication was discontinued and hypoglycemia resolved spontaneously without the need for pancreatic surgery.

Data were analyzed by SPSS 22.0 (©IBM-SPSS), with differences in frequencies of categorical variables being assessed by chi-squared tests and those between continuous variables being assessed by Mann–Whitney tests. The association of FPs with severity of CHI was determined by a backward-stepwise logistic regression model. The study was approved by the local research ethics committee.

## Results

### Prevalence, Types, and Persistence of FPs

In this cohort (*n* = 83, 50 males), 28 (34%) children (males:females = 21:7) with CHI were identified to have FPs at diagnosis. CHI characteristics in those with and without FPs have been provided in Table 1. Most CHI children with FPs presented early at a median age (range) of 1 (1–365) days, but two children presented between 3 and 4 months and three children presented between age 7 and 12 months. CHI mutations were present in 17 (63%) children. At presentation, hypoglycemia was severe enough to require intravenous glucagon infusion treatment in 14 (52%) children. All children were treated with diazoxide, with dosage starting at 5 mg/kg/day and escalating to a maximum of 15 (7–20) mg/kg/day. In the cohort with FPs, 6 (22%) children achieved spontaneous resolution of hypoglycemia, while a further 6 (21%) children required octreotide for medical treatment of persistent CHI. Focal lesions were present in five (19%) children, four of whom were treated by focal lesionectomy and one child did not require surgery. Subtotal pancreatectomy was performed in 10 (35%) children. All families with FPs required clinical psychology support at initial presentation and at the 6- and 12-month follow-up appointments. Such support was non-structured and the interactions were not objectively assessed.

**Table 1:** **Differences between groups of children with and without feeding problems (FPs): continuous variables are represented as median (range) values, while categorical variables are represented as percentages**.

	Feeding problems (*n* = 28)	No feeding problems (*n* = 55)	*p* value
Age at presentation (days)	1 (1–365)	1 (1–630)	0.21
Insulin at diagnosis (mU/l)	18.5 (2.5–110.0)	11.2 (2.2–132.0)	0.32
CHI mutations (%)	17 (63.0)	15 (26.8)	0.002*
Maximal dose of diazoxide (mg/kg/day)	15.0 (7.0–20.0)	6.1 (4.5–21.0)	<0.001*
Octreotide treatment (%)	6 (22)	0 (0)	<0.001*
Glucagon infusion (%)	14 (51.9)	5 (8.9)	<0.001*
Subtotal pancreatectomy (%)	10 (35)	0 (0)	<0.001*
Focal CHI (%)	5 (18.5)	3 (5.4)	0.06
Spontaneous resolution (%)	6 (22.2)	32 (58.2)	0.002*

*p values <0.05 were considered significant.

**Figure 1:**
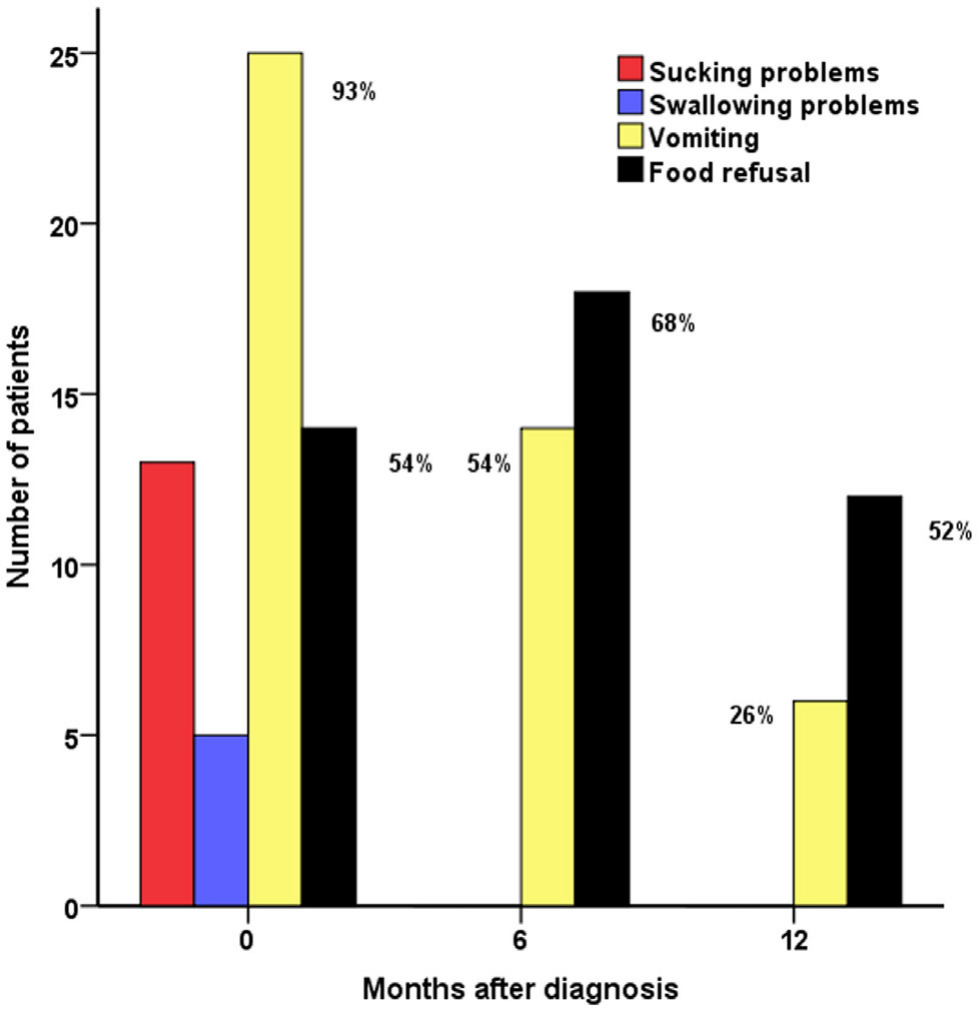
**Feeding problems (FPs) in CHI patients (*n* = 28): sucking problems, swallowing problems, vomiting, and food refusal at diagnosis and at 6 and 12 months in follow-up assessments**.

All children with FPs at the time of diagnosis had at least one of the following: sucking problems (*n* = 14, 50%), swallowing problems (*n* = 4, 15%), vomiting (*n* = 26, 93%), and food refusal (*n* = 15, 54%) (Figure 1). Most children (*n* = 26, 93%) required antireflux treatment with antacids, gelling agents or feed thickeners (*n* = 7, 25%), ranitidine (*n* = 19, 68%), domperidone (*n* = 25, 89%), omeprazole (*n* = 13, 46%), and erythromycin (*n* = 4, 14%). Nasogastric tube feeding was commenced in the majority of children with FPs (*n* = 21, 75%) to aid enteral feeding to achieve normal glucose status. Gastrostomy tubes (*n* = 21, 75%) were inserted after 105 (20; 288) days of nasogastric tube feeding to facilitate nutritional intake. Insertion of gastrostomy in the child with the longest duration of nasogastric tube feeding (288 days) was delayed because of parental choice. In one child, gastrostomy was inserted pre-emptively at the time of pancreatic surgery.

Sucking and swallowing problems were present at the time of diagnosis but were absent during follow-up. At the 6-month assessment, vomiting was still prevalent in a majority (*n* = 15, 54%), with subsequent reduction at 12 months (*n* = 7, 26%) (*p* < 0.001, test for difference across 0, 6, and 12 months). Food refusal was present in a significant proportion of children at diagnosis, at follow-up at 6 months (*n* = 19, 68%), and at 12 months (*n* = 14, 52%), indicating long-term persistence of the problem (*p* = 0.14 for difference).

In children with FPs, weight SDS was similar at presentation, 6 and 12 months in follow-up [median (range) 1.8 (−2.2 to 8.3) vs. 1.30 (−2.3 to 4.3) vs. 1.7 (−2.3 to 5.1), *p* = 0.61 for difference between diagnosis and 6 months, *p* = 0.08 for difference between 6 and 12 months and *p* = 0.41 for overall difference], suggesting appropriate nutritional intake despite FPs. Height was also similar with no difference observed between 6 and 12 months [0.9 (−1.0 to 3.7) vs. 0.6 (−1.0 to 3.5), *p* = 0.32].

### Risk Factors and Associations with FPs

In contrast to the group of children without FPs, those with FPs required higher maximal diazoxide dose and had a higher frequency of glucagon infusions, indicating greater severity of disease (Table 1). Diazoxide itself was not the cause for FPs; it was evident by the observation that FPs was persistent in those in whom diazoxide was discontinued (*n* = 9, 53%). Further, the maximum dose of diazoxide was not correlated with the presence of food refusal either at diagnosis (*p* = 0.98), 6 months (*p* = 0.71), or at 12 months (*p* = 0.22), indicating the absence of a diazoxide dose-dependent effect on the prevalence of FPs, either in the short- or long term. Therefore, the association of FPs and diazoxide implies an indirect correlation of FPs with the severity of CHI. Likewise, FPs predated the introduction of octreotide, which was commenced following unresponsiveness or adverse events on diazoxide. Therefore, it is unlikely that FPs were causally related to octreotide. This is further supported by the observation that FPs persisted after octreotide was discontinued.

The group with FPs also had a higher frequency of genetic mutations, a higher frequency of children requiring medical treatment with octreotide, and a higher proportion of children requiring pancreatectomy, indicating more severe CHI. Not surprisingly, few children in this group achieved spontaneous resolution.

Markers suggestive of greater severity of CHI disease were entered into a backward logistic regression model (*R*^2^ = 0.50, *p* < 0.001), using and adjusting for variables in Table 1 (with the addition of gender, gestation, and birth weight). Diazoxide and octreotide were excluded from the regression as FPs predated and postdated the start and stop times for treatment to bear temporal association. FPs were most likely to be associated with the presence of K_ATP_ channel gene mutations as expected [odds ratio, OR (95% confidence intervals, CI) 6.16 (1.1–36.3), *p* = 0.04] and the use of glucagon infusion [OR (CI) 28.13 (2.6–300.1), *p* = 0.006]. In those with FPs, glucagon was administered in 6 (16%) children who achieved spontaneous resolution in contrast to 13 (30%) children who had persistent disease (*p* = 0.19 for difference). Therefore, cumulative dose of glucagon was not correlated with FPs.

In the group with FPs, food refusal at diagnosis was not correlated with birth weight, gestation, age at presentation, K_ATP_ channel gene mutations, diagnosis of focal CHI, requirement of glucagon infusion, or the achievement of spontaneous resolution. Food refusal at 6 months (*R*^2^ = 0.54 for model, *p* = 0.001) was less likely in those who achieved spontaneous resolution [OR 0.1 (0.1–0.8), *p* = 0.04] and had a focal lesion [OR 0.1 (0.1–0.2), *p* = 0.004], the latter retaining significance at 12 months in a similar model [OR 0.1 (0.0–0.7), *R*^2^ = 0.42, *p* = 0.002].

To test the possibility that nasogastric tube feeding was detrimental to oral feeding, age at commencing and stopping nasogastric tube feeding was tested for correlation with the frequency of FPs at 6 and 12 months. There was no difference in FPs frequency for age at commencing or age at stopping nasogastric tube feeding at 6 months (*p* = 0.57, *p* = 0.28) or 12 months (*p* = 0.33, *p* = 0.30), respectively. Therefore, the introduction and persistence of nasogastric tube feeding were not directly implicated in FPs. The presence of gastrostomy feeding was also tested against food refusal at 12 months, but there was no association (*p* = 0.24). This suggests that late, persistent food refusal was not related to gastrostomy insertion after diagnosis. The requirement for persistence of nasogastric tube feeding has been supported by two case illustrations in Tables 2 and 3.

**Table 2:** **Case illustration**.

Patient #14 carrying a GCK mutation had diazoxide-responsive CHI. A nasogastric tube had been inserted at birth to support feeding and manage hypoglycemia. Although glycemic stability was achieved, the patient continued to experience increasing problems with vomiting and oral feed intolerance, in spite of antireflux medication and variations in milk formulations, thereby requiring persistent nasogastric tube feeding as a reliable source of nutritional intake. Due to the reliance on nasogastric tube feeding, a gastrostomy tube was inserted at the age of 157 days. The patient required regular speech and language therapy and dietetic input and at the age of 2.5 years gradually improved oral intake, although occasionally reliant on gastrostomy feeding. This case illustrates the practical difficulty with weaning nasogastric tube feeding in some children with CHI complicated by FPs.

**Table 3:** **Case illustration**.

Patient #19 who had diffuse CHI with compound heterozygous mutations in ABCC8, being unresponsive to diazoxide and octreotide, required carbohydrate supplementation in feeds to achieve glycemic stability. With increasing oral aversion, he became reliant on nasogastric tube feeding. At the time of subtotal pancreatectomy, a gastrostomy tube was inserted. In the postoperative period, the patient’s FPs worsened with difficulty in maintaining glycemic control. The patient achieved improved glycemic stability with a second near-total pancreatectomy procedure at the age of 389 days. Since then, the patient improved gradually with oral intake and eventually stopped relying on gastrostomy feeding at the age of 7 years. This case illustrates the relationship between hyperinsulinism and FPs in that delayed resolution of CHI is associated with persistence of FPs.
In contrast, patient #17 had inherited a paternal heterozygous mutation in ABCC8 and had a focal lesion on PET-CT scanning. A nasogastric tube had been inserted shortly at diagnosis to maintain glucose control. In the time leading up to focal lesionectomy surgery, the patient developed oral feed intolerance. At pancreatic surgery at the age of 90 days, a gastrostomy tube was inserted. In the postoperative period, glycemic stability was achieved. At the same time, oral feed tolerance returned with no further reliance on gastrostomy feeding. Patient #17’s case contrasts to that to patient #19, illustrating the possibility that presence and resolution of FPs are associated with the persistence and cure of CHI.

## Discussion

We have described the prevalence and persistence of FPs in a significant proportion of children with CHI. Vomiting and food refusal are major problems, which may occur at diagnosis but are more prominent later in the course of the disease. Analysis of factors characterizing CHI at diagnosis and review at 6 and 12 months suggests that CHI disease severity may play a causal role. The use of medications, nasogastric tube feeding, and gastrostomy devices are unlikely to be individually responsible for causing FPs.

There have been no comparative studies reporting on the prevalence of FPs, although a recent study noted that 19 (45%) out of 42 patients with severe CHI, of whom 79% required subtotal pancreatectomy over a 15-year period, had food aversion (6). While FPs is well recognized in CHI, causal and associated factors have not been analyzed. It is commonly assumed that FPs may be secondary to treatment, such as diazoxide (metallic after taste) and octreotide (gut dysmotility), or aggravated by nasogastric tube feeding and subsequent lack of oral exposure. However, in our study, we have shown that FPs is less likely to be influenced by medication (diazoxide and octreotide) and the introduction of nasogastric tube feeding than by the severity of illness. The correlation of glucagon administration with FPs is likely to reflect severity of CHI, although the possibility of glucagon directly suppressing feeding behavior through its effects on the central nervous system cannot be excluded (10). The observation that glucagon treatment was associated with FPs regardless of cumulative dose argues against a dose–response effect and hence less likely a treatment side effect. Further evidence for the link of FPs with severity comes with the observation of improvement in FPs with resolution of CHI, as in those with spontaneous resolution and those in the postfocal lesionectomy period. However, the study did not address the temporal correlation between resolution of CHI and the resolution of FPs, which could be investigated by longitudinal follow-up studies examining the natural history of CHI. Nonetheless, no child with normal feeding developed late onset FPs, reinforcing the correlation of severity of CHI at presentation and FPs.

All children who required nasogastric tube feeding for persistent FPs had gastrostomy insertion. Therefore, it appears from our cohort that gastrostomy feeding is more likely in children who have persistent severe FPs, and that early gastrostomy insertion may be necessary in the clinical management of CHI complicated by FPs.

The prevalence of K_ATP_ channel gene mutations, diazoxide responsiveness, and rates for surgery is broadly commensurate with other CHI cohorts (7). Although our cohort of 28 children with FPs is relatively small, they have been well characterized, both from a CHI perspective and from a feeding/dietetic perspective, with formal evaluation at 6 and 12 months after diagnosis. Further, we have recruited a relatively large group without FPs to enhance the strength of associations suggesting severity of hyperinsulinism as a probable causal agent.

We did not specifically evaluate the neurological status of patients in this cohort, in contrast to a previous study (6), but had excluded patients with severe neurological abnormalities likely to adversely influence feeding. It is possible that significant neurological adverse outcomes secondary to neuroglycopenia may have caused FPs in some individuals. However, at 12 months postdiagnosis, no child recruited to this study had evidence of neurologic injury severe enough to cause FPs. Although not assessed, children with mild neurodevelopmental impairment were likely to be present both in those with and without FPs, suggesting an unlikely role for brain damage as a cause for FPs in our short-term assessment. Nonetheless, it would be important to assess for mild neurodevelopmental impairment in the long-term follow-up of children with FPs.

While examining for risk factors influencing FPs, we have not assessed if hyperglycemia may have played a part in the causation of FPs. It is possible that FPs occurred in this cohort due to inadvertent hyperglycemia from overenthusiastic glucose supplementation in some individuals, although there is no evidence that hyperglycemia in neonates causes FPs. In older children with type 1 diabetes, vomiting or food refusal is not commonly described, although gastroparesis can occur in 10% individuals (11). While electrogastrography studies in type 1 diabetes children suggest early onset of dysmotility problems due to acute hyperglycemia, there is no strong evidence for hyperglycemia to cause food refusal behavior in younger children with CHI.

All children and families who had FPs required clinical psychology support as part of routine clinical management. The psychology support addressed FPs in addition to other issues related to CHI, i.e., stress related to disease, long duration of treatment, and hospital stay. However, the study did not quantify the psychological impact on the family through inventories and questionnaires. The design did not incorporate strategies to investigate and ameliorate psychological concerns specifically arising from FPs in the context of CHI. However, considering that children with FPs required psychological support, future studies should explore FPs-specific psychological outcomes.

In our study, we are aware that the strength of association of FPs with factors marking severity in subgroup analyses may be weak. Nonetheless, the reduction of food refusal prevalence after spontaneous resolution of CHI and focal CHI surgery is compelling evidence to suggest a role for persistent hyperinsulinism in the pathogenesis of FPs. It is recognized that insulin can be an appetite stimulant in normal individuals, mediated by hypoglycemia. However, it has also been recognized that gastric motility can be reduced in adult healthy volunteers by the introduction of relatively large doses of insulin (12). Recent work suggests that insulin may also reduce appetite and feeding through signaling mechanisms in the brain (13), with further evidence that insulin may suppress appetite by anorexigenic mechanisms in the hypothalamus, either activating mammalian target of rapamycin 1 (mTORC1) (14) or suppressing ghrelin-induced calcium signaling in neuropeptide Y neurons (15). It is possible that severe hyperinsulinism in children with CHI may play a causal role in the pathogenesis of persistent FPs, consistent with observations that insulin signaling in the brain may regulate feeding behavior in animals (16).

## Conclusion

FPs can occur in children with CHI and may persist until 1 year after diagnosis, particularly as food refusal. The cause for FPs is not clear but severity of CHI is a consistent association. Further studies are required to evaluate the longer term natural history of FPs and to investigate how insulin alters feeding behavior and whether prompt treatment of hyperinsulinism may prevent FPs in children with CHI.

## Author Contributions

All authors contributed to the study and writing of the manuscript. PC, KC, MS, and MD conceived the design of the study. IB, LF, and HA collected data with the assistance of LB, LR, RC, RP, MS, SE, LP, and PC. Data were analyzed by IB, LF, and PC. The draft manuscript was prepared by IB and LF with critical review by PC and agreement with other authors. The final manuscript was prepared by IB and PC.

## Conflict of Interest Statement

The authors have no conflict of interest that could prejudice the impartiality of the research reported.
